# Interactive framework for Covid-19 detection and segmentation with feedback facility for dynamically improved accuracy and trust

**DOI:** 10.1371/journal.pone.0278487

**Published:** 2022-12-22

**Authors:** Kashfia Sailunaz, Deniz Bestepe, Tansel Özyer, Jon Rokne, Reda Alhajj

**Affiliations:** 1 Department of Computer Science, University of Calgary, Calgary, Alberta, Canada; 2 Department of Computer Engineering, Istanbul Medipol University, Istanbul, Turkey; 3 Department of Computer Engineering, Ankara Medipol University, Ankara, Turkey; 4 Department of Health Informatics, University of Southern Denmark, Odense, Denmark; Politechnika Slaska, POLAND

## Abstract

Due to the severity and speed of spread of the ongoing Covid-19 pandemic, fast but accurate diagnosis of Covid-19 patients has become a crucial task. Achievements in this respect might enlighten future efforts for the containment of other possible pandemics. Researchers from various fields have been trying to provide novel ideas for models or systems to identify Covid-19 patients from different medical and non-medical data. AI-based researchers have also been trying to contribute to this area by mostly providing novel approaches of automated systems using convolutional neural network (CNN) and deep neural network (DNN) for Covid-19 detection and diagnosis. Due to the efficiency of deep learning (DL) and transfer learning (TL) models in classification and segmentation tasks, most of the recent AI-based researches proposed various DL and TL models for Covid-19 detection and infected region segmentation from chest medical images like X-rays or CT images. This paper describes a web-based application framework for Covid-19 lung infection detection and segmentation. The proposed framework is characterized by a feedback mechanism for self learning and tuning. It uses variations of three popular DL models, namely Mask R-CNN, U-Net, and U-Net++. The models were trained, evaluated and tested using CT images of Covid patients which were collected from two different sources. The web application provide a simple user friendly interface to process the CT images from various resources using the chosen models, thresholds and other parameters to generate the decisions on detection and segmentation. The models achieve high performance scores for Dice similarity, Jaccard similarity, accuracy, loss, and precision values. The U-Net model outperformed the other models with more than 98% accuracy.

## Introduction

The novel coronavirus named Severe Acute Respiratory Syndrome Corona-Virus 2 (SARS-CoV-2) was reported in Wuhan, China for the first time in December 2019 appearing as pneumonia cases with unknown cause [1, 2]. It is a positive-sense single stranded RNA virus that causes respiratory, neurological, hepatic and other health issues [3]. The World Health Organization (WHO) declared it a ‘Public Health Emergency of International Concern (PHEIC)’ on January 30, 2020 due to the severe and rapid outbreak. On March 11, 2020, WHO declared iCOVID-19 to be a ‘Global Pandemic’ after the cases increased all over the world with 118,000 cases in 114 countries, and 4,291 deaths [4, 5].

On February 25, 2022, WHO reported 430,257,564 confirmed cases of COVID-19, including 5,922,049 deaths [6]. The SARS-CoV-2 virus have mutated over time like any other viruses. The mutations have impacted the various virus properties like disease severity, the speed and ease of spread, diagnostic tools, vaccine performance, etc. creating new variants of the disease. The few variants of concern (VOC) and the variants of interest (VOI) are ‘Alpha’, ‘Beta’, ‘Gamma’, ‘Delta’, ‘Lambda’, ‘Mu’, and ‘Omicron’. Their earliest samples were detected in United Kingdom, South Africa, Brazil, India, Peru, Columbia, and later in multiple other countries [7]. Generally, nucleic acid-based test approaches such as polymerase chain reaction (PCR) is the most reliable for virus detection and the reverse transcriptase-PCR (RT-PCR) is considered as the ‘gold standard’ for SARS-CoV-2 virus detection [8]. As the RT-PCR test is time-consuming and there are not enough test kits available, chest imaging approaches can be a helpful alternative for faster Covid-19 detection. Various chest imaging tools such as chest X-rays, chest CT scans and lung ultrasounds can be used for Covid-19 detection, severity analysis and diagnosis [9]. The availability of at least one or more of these chest imaging tools provides a more affordable diagnosis at lower cost compared to the RT-PCR kits. Further, image processing is faster compared to the RT-PCR results.

Chest image processing for Covid-19 detection is focused on identifying any abnormalities in the chest images [10]. Some common indication of Covid-19 in chest images are—different types of opacity (i.e., subpleural curvilinear opacity, reticulonodular opacity, ground-glass opacity, etc.), septal thickening, crazy-paving appearances, consolidation, cavitating lesions and various irregular shapes (i.e., tree-in-bud, halo sign, etc.) [11]. The analysis of chest images together with RT-PCR test results help healthcare professionals with Covid-19 diagnosis. Hence, applying AI tools to automate chest image analysis for identifying Covid-19 patients and for segmenting the infected regions could speed up the diagnosis while reducing the manual workloads of healthcare workers [12]. This had encouraged researchers to apply various machine learning (ML), deep learning (DL) and transfer learning (TL) techniques on chest images for automatic Covid-19 detection, segmentation, severity prediction, diagnosis and patient monitoring [13]. In this paper, we therefore propose a DL-based Covid-19 detection and segmentation system with a web-based application which has a user interface to access the system that can classify Covid-19 patients and segment the infected lung regions from chest imaging.

The proposed system framework includes a web-based application that can be used to upload and evaluate chest CT images in various formats (i.e., PNG, JPG, DICOM, etc.) using one of three DL models (namely, Mask R-CNN, U-Net, U-Net++) to be chosen by a user who can also set various thresholds and parameters. The framework pre-processes an image to extract the lungs part of the image by removing the background and then uses the lungs image to detect and segment the Covid infected regions. The interface shows the infected regions in the lungs together with other patient information, and the ratio of the infection in the lungs helps in specifying the severity of the infection. The DL models use a combination of two annotated datasets to train, evaluate and test each model individually. Three versions of the Mask R-CNN model with three different epoch values (i.e., 40, 60, and 80), basic U-Net, and U-Net++ performance scores and segmentation outputs were compared and the comparisons provided some directions for choosing the best model for image processing. The interface also provides options for medical professionals to submit their feedback on the outcome (segmentation/annotation) produced by the system. The feedback is then added to the dataset and the updated dataset is used to refine the models for improved future image processing. Although some researches on Covid-19 infection segmentation with DNNs applied the feedback process from medical professional [14], they applied the feedbacks (i.e. correct or incorrect) only on a few random images as a contribution to future training. Our proposed system instead provides a complete framework for medical professionals to provide detail feedbacks including drawing the correct segmentation and submitting the resulting image to the system for future training.

There are existing systems that can detect Covid-19 from medical images, segment infectious parts of the lungs, or can both detect and segment the infections. Very few of the existing works mention automated report generation or feedback incorporation from medical professionals. Our proposed system provides a complete application for Covid-19 detection, infection region segmentation, and feedback processing from medical professionals to improve future training for the process using medical images. The novelty of our proposed system is it will create a web application by combining all these tasks in a single system with a user friendly interface that can help medical professionals by providing the detection and segmentation results as a pre-screening with multiple deep learning algorithm and thresholding choices and by taking inputs from them with more accurate segmentation to improve the training process for future usage of the system. The major contributions of this research is a proposal for -

1. generating a web application for Covid-19 lung infection detection and segmentation from medical images for clinical and research usage,
2. evaluating lungs images (i.e., CT images) to detect Covid-19 infected regions by providing five alternative DL models—three variations of Mask R-CNN (Mask R-CNN 40, Mask R-CNN 60, and Mask R-CNN 80), U-Net, U-Net++ that the user can choose from,
3. detecting accurate infection regions by fine tuning model parameters (i.e., thresholds) directly using the web application,
4. allowing medical professionals to provide feedback to fix any potential misdetection of the infected regions by allowing them to draw the boundaries for the infection regions, using the feedback for self-tuning, and hence improve the performance of the models.

The rest of the paper is organized as follows. The related works are briefly covered in the next Section. The methodology, experimental setup and results follow the related works. Finally the paper is concluded with a conclusion.

## Related works

Covid-19 emerged at the end of 2019 and researchers are still trying to find accurate detection and diagnosis systems. A large amount of research has also been published on Covid-19 analysis with the hope to cope with its severity. However, only a few researchers have reviewed and summarized the existing works to provide an overview of the current progress and future research directions. Alafif et al. [15] recently reviewed existing researches for Covid-19 detection, segmentation, and diagnosis using chest imaging (i.e., X-rays and CT scans) and sound, available AI tools, drugs and vaccines. They discussed various ML, DL and hybrid models used for Covid analysis while listing the challenges regarding the scant data, the innovative AI, the lack of medical and other human resources. Similarly, some other recent survey papers reviewed Covid-19 researches using medical images (mostly chest X-ray and CT) for detecting, segmenting, classifying, monitoring and diagnosing Covid [16–22]. ML, DL, TL and hybrid models like SVM, RF, DT, KNN, NB, LightGBM, XGBoost, AdaBoost, Bagging, ensemble classifiers, ResNet, Inception, InceptionResNet, IRRCNN, ShuffleNet, NASNetLarge. GoogleNet, CNN, AlexNet, VGG, SqueezeNet, SENet, Xception, CapsNet, autoencoder, MobileNet, DenseNet, attention, U-Net, U-Net++, GAN etc. were applied to medical images and most of them achieved high performance on the few available or customized Covid datasets. Most of them presented the classification tasks to classify Covid patient data from non-Covid patient data. Non-Covid patient data included healthy people, people with pneumonia, and some researches included more divisions of pneumonia classifying it into viral or bacterial pneumonia.

Some other recent survey papers, e.g., [23–25], discussed researches using not only chest X-ray/CTs, but also other clinical data, lab tests, textual information, demographic data, medication, statistics, etc. for multiple tasks like Covid-19 detection, segmentation, classification, prediction, forecasting, diagnosis, screening and assessment. They mentioned the recent works using ML models like RF, SVM, LR, XGBoost, KNN, K-means, LR, DT, ANN etc. and DL models such as CNN, VGG, ResNet, Inception, RNN, DenseNet, LSTM etc. and hybrid models on Covid patient data and achieved more than 90% accuracy. Most of these reviews also discussed the challenges, scope and possible future directions for Covid-19 researchers. Some researchers also applied heuristic-based methods for Covid-19 medical image analysis. Hamza et al. [26] used moth flame optimization on a CNN-LSTM based Covid-19 classification model on chest X-rays. Chest X-rays were used to enhance the dataset with data augmentation and a CNN-LSTM model was implemented for deep feature extraction. Features were fused and the best features were selected by a moth flame optimization heuristic algorithm to classify the images into normal, Covid-19, lung-opacity, normal pneumonia, viral pneumonia and tuberculosis classes.

Most recent research efforts on Covid-19 image datasets used DL or TL, and a few researchers have applied some conventional image processing approaches to the classification problem with comparable performance. Although most research on Covid-19 segmentation applied DNNs for lung segmentation as a part of pre-processing for Covid-19 classification to extract only the lung regions from the medical images [27, 28], more recent researches are focusing on segmenting the infections and lesions from lungs using DNN and hybrid models. Sundaram et al. [29] recently proposed a two stage DL framework by combining residual SqueezeNet and SegNet (RSqz-SegNet) models to detect Covid images and segment opacities, granulomas and subtle infections of Covid patients. The model detected Covid patients from viral pneumonia and bacterial pneumonia patients with more than 99% accuracy. The segmentation network was able to segment the infection regions with more than 82% accuracy for two different datasets. The segmentation framework outperformed six other ML, DL and hybrid models containing VGG, ResNet, SVM, SqueezeNet, etc. by 1% to 9% and was able to achieve comparable segmentation performance as U-Nets. Sait et al. [30] recently proposed a multimodal framework called Ai-CovScan for Covid-19 diagnosis system that utilized deep transfer learning named CovScanNet on breathing sound, chest X-ray and a rapid antigent test. The system analyzed breathing sound of a patient and generates a spectogram video which is then segmented to generate spectogram images. The chest X-ray and spectogram images were individually pre-processesd to train an Inception-V3 DNN for feature extraction and a multilayer perceptron (MLP) was used for classification, respectively. Sound was classified into normal, fine crackles, coarse crackles and wheezes whereas x-ray images were classified into normal, Covid-19, viral pneumonia and bacterial pneumonia. They also proposed a smartphone application for Ai-CovScan system to enable e-diagnosis to help the healthcare system. The proposed model provided 80% accuracy for Covid-19 detection from breathing sound and 99.66% accuracy from chest X-ray while decreasing the false-negatives. Oulefki et al. [31] proposed a Covid-19 lung infection segmentation and measurement model with image-dependent multilevel thresholding to minimize over segmented regions. A new masking algorithm was used which contains multiple thresholding, filtering and entropy calculation on the image histogram to generate masks for the infected regions of the lungs. The proposed segmentation model achieved more than 98% accuracy with 0.714 dice coefficient score that outperformed 9 popular DNNs and similar medical image segmentation models. Visual representation and assessment were performed on the segmented images to show the infected area and severity level of infections with different colors. The novel and simple multi-level thresholding algorithm was able to quite accurately extract the infected lung regions for Covid-19 patients.

Due to the high performance of DNN models in medical image analysis, most of the recent research efforts have focused on DL and TL methods. A new Covid-19 image dataset with 433 annotated chest CT images collected from 82 COVID-19 patients has been recently provided in [32]. They also proposed a DL segmentation model for segmenting the Ground Glass Opacity (GGO) and Consolidation (C) infection regions. They trained and tested their model with four datasets (including their own). Their model was a FCNN which was an encoder-decoder-based network with encoding path, transition layers, context perception boosting module, and decoding path for image segmentation. The proposed model was able to achieve high performance scores (i.e., around 0.80 dice score, 0.99 specificity, etc.) in detecting GGO and C pixels. Aleem et al. [33] used Mask R-CNN for Covid-19 infection area detection from chest CT scans. They provided a basic UI for storing, accessing and analyzing patient data while providing reports for the patients. They used ResNet50 and ResNet101 as Mask R-CNN backbones individually to segment the area of the infection and provided the ratio of the infected area in the lungs. After segmenting the objects with Mask R-CNN, 50 ROIs were kept and finally the highest probability ROI bounding boxes were shown as results to highlight the infections. Based on the infection ratio (i.e., intensity), images were classified as ‘Mild’ or ‘Alarming’, and the UI provided for different types of users showed the bounding box of the infection with the intensity level. The UI also generated a prediction graph for each patient for 3 or more days using the AI models. Covid-ct-mask-net [34], a variation of the Mask R-CNN model was recently proposed for detecting and segmenting two types of Covid related lesions from lungs CT images. ResNet and Feature Pyramid Net (FPN) were used as the backbone of the model, and their output was used by a Region Proposal Net (RPN) to create the bounding boxes for the detected ROIs. The model was trained with 3000 Covid, pneumonia and control images; it was evaluated with more than 21192 different images. It outperformed seven other DL model based Covid detection and segmentation systems with 91% accuracy and more than 90% sensitivity. Another research on Covid-19 chest X-rays used Mask R-CNN for mucus plug blockage detection in [35]. A CNN model was used for binary classification to identify X-rays of Covid patients. The Covid positive X-rays were then used for detecting and segmenting mucus plug blockage with Mask R-CNN.

Among various DNNs, U-Net and its variations are popular with medical image analysis for their high accuracy rates. Hence, researchers have been using U-Net models for Covid-19 detection, classification and segmentation from image datasets. A soft attention based U-Net was proposed in [36] for Covid-19 lesion region segmentation from chest CTs. Spatial, color and noise augmentation were applied to extend 3 datasets and soft attention was applied at U-Net layers. More implicit features were extracted that achieved more then 98% accuracy and outperformed basic U-Net variations. CHS-Net [37], a hierarchical segmentation model for Covid-19 CT was used by extracting semantic data with two cascaded residual attention inception U-Net (RAIU-Net). Applying hybrid pooling function and spectral attention module to skip connections improved the segmentation performance for both the lung segmentation and finally the infection region segmentation. Voulodimos et al. [14] incorporated a feedback option in their few-shot U-net model for Covid-19 infection segmentation. The U-Net model was trained for infection segmentation and a medical professional randomly selected few incorrectly evaluated outputs to check and provided their feedbacks. The system then used these feedbacks for the next training phase.

Degerli et al. [38] proposed a Covid-19 detection and segmentation model with U-Net, U-Net++ and DLA while providing one of the largest Covid X-ray datasets called ‘Qata-COV19’. Twenty four combinations of these 3 DNNs with 4 pre-trained encoders DenseNet-121, CheXNet, Inception-v3 and ResNet-50 in frozen and non-frozen modes were applied on the X-rays to show that U-Net and U-Net++ with DenseNet-121 achieved more than 99% detection and segmentation accuracy. Another DL model using U-Net++ for Covid segmentation and detection is provided in [39] with a basic UI. They collected chest CT of Covid patients from Renmin Hospital of Wuhan University for developing their automated system. They validated their system using an external test on Qianjiang Central Hospital data to evaluate its robustness. They included data from control patients to balance the datasets and to train the model appropriately. They used a U-Net++ model with a pre-trained ResNet-50 backbone for Covid infection detection and segmentation. The system achieved accuracy values in the range 95.24% and 98.85% for images of the training, validation and testing phases. They created a basic UI that allowed the users to upload CT image and submit them for diagnosis of Covid-19 pneumonia.

Saeedizadeh et al. [40] proposed a modified U-Net model called ‘COVID TV-Unet’ for Covid infection (i.e., ground glass regions) segmentation from chest CT images. A modified loss function with 2D-anisotropic total-variation was introduced for connectivity-promoting regularization of a U-Net model. The total-variant loss was computed and added to the binary cross entropy (BCE) loss to evaluate the total loss function. Various combinations of split thresholds, loss function, and optimizers were applied on the datasets. Then, the best parameters were chosen from the results for the proposed system. The proposed TV-Unet model was compared with U-Net+, Inf-Net, Semi-Inf-Net on Covid chest CT images; it outperformed them with 0.801 dice score. Another variation of U-Net named ‘CARes‐UNet (content-aware residual UNet)’ was proposed in [41] for lesion segmentation from Covid chest CT images. The residual network was used for improving the segmentation performance. The content aware upsampling module improved the performance and optimized the computation cost. An advanced optimizer ‘Ranger’ was used to decrease the convergence time of the model and a semi-supervised approach was applied to overcome the limitations of limited pixel-level labeling. CARes-UNet achieved 0.731 dice score whereas the semi-CARes-UNet performed better by achieving a dice score of 0.776. They outperformed most of the nine other popular DL models for Covid lesion segmentation. SD-UNet [42], a modified U-Net framework, was proposed with the squeeze-and-attention (SA) and dense atrous spatial pyramid pooling (Dense ASPP) modules. The network used lungs masks to extract only the lungs from the images. The model was trained with the lungs regions and the ground truth containing the infection regions. The combined advantages of U-Net, attention network and dense ASPP enabled the proposed model to outperform similar basic or modified U-Net and U-Net++ models. SD-UNet also provided a more specific infection region segmentation with 94% accuracy for GGO and C lesions creating the possibility of practical application of the model in real-time medical image based systems.

Attention Gate-Dense Network-Improved Dilation Convolution-UNET (ADID-UNET) [43] is another U-Net variation which was proposed recently for Covid infection segmentation from lungs CT scans. A dense network combining convolution layers, transition layers, pool functions and dense blocks was used in the U-Net instead of a max pool layer to extract dense features. An improved dilation convolution was used to refine and extract more specific edge features for small infection areas and the attention model improved the prediction accuracy by focusing on infection ROIs. ADID-UNET performed better than similar U-Net variations and other DNNs used on Covid CT scans for infection segmentation with more than 97% accuracy and 0.80 dice score. A multi-task multi-instance deep network (*M*^2^ UNet) was proposed by Zhao et al. [44]. It segments the infectious region from Covid CT images while detecting the severity level of the infection. They trained their U-Net model on 2D patches generated from 3D CT images to extract image features. The features were then used to classify the images as severe or non-severe cases of Covid. They also provided a visualization for the images to show the infection regions of both lungs with various colors. The proposed model achieved 0.985 accuracy with 0.785 DCC; it outperformed similar U-Net, U-Net+ and Res-Net models.

Researchers used various types of machine learning, deep learning, transfer learning and hybrid models for Covid-19 detection, classification, and segmentation from different types of inputs. As expected, the deep learning models performed better compared to the other systems and were able to achieve high accuracy. But Covid-19 research efforts need more data, correctly annotated data and experiments to generalize the results of those models, and to apply them in real time systems. Our research proposed a system to minimize these limitations by providing a deep learning base Covid-19 detection and segmentation system with a web application for medical professionals to check the output and provide feedback to refine the system performance and update the annotation for chest medical images.

## Methodology

**Fig 1:**
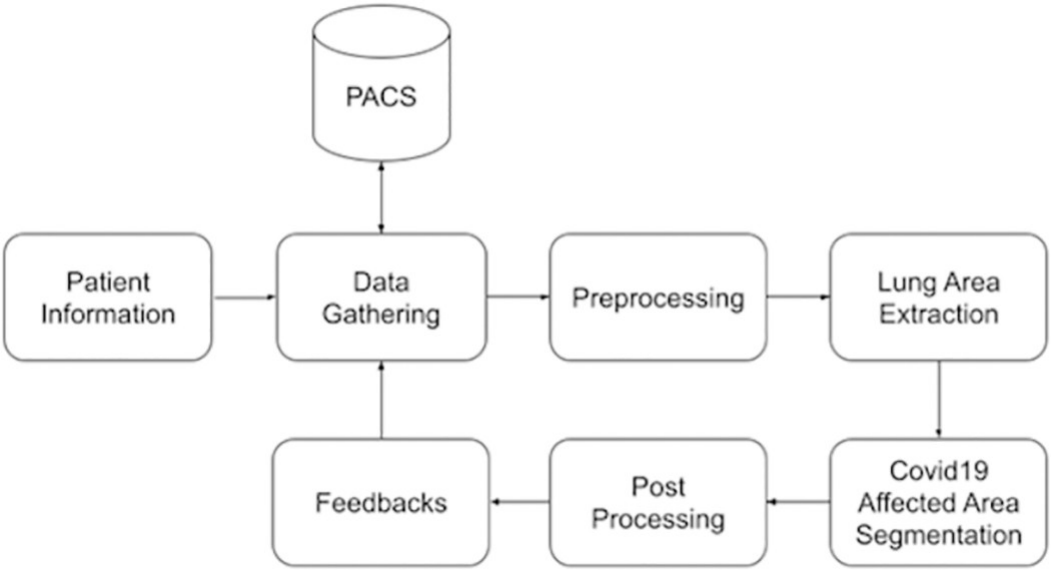
Block diagram of the proposed system.

A block diagram of the proposed DNN-based Covid-19 detection and segmentation model is shown in Fig 1. The complete framework consists of two main parts—(i) the web application and user interface with feedback facility, and (ii) the segmentation with DNNs. The system can receive input from both healthcare professionals and hospitals. By integrating the Picture Archiving and Communication System (PACS), the system can connect directly to the present imaging systems at any given hospital, and help its users in instantly assessing patient’s conditions. The system can be accessed by users (i.e., healthcare professionals) and the system administrator. The system is also adaptive to the user’s feedback. During each evaluation, experts are given the capability to criticize the output produced by the system and give their feedback by marking what they consider as misclassification. This will help the system in self tuning by considering the provided feedback in improving its learning process to achieve higher accuracy. As a result of this feedback learning process, the system is expected to stabilize where the amount of feedback will decrease leading to higher accuracy satisfactory and appreciated by domain experts. The admins can only see feedback and provided labels, which can be compared to refine the results later on.

The process starts when the users enter the patient information and evaluation parameters. The system gathers the required CT images from PACS and applies the pre-processing steps to convert the images into the appropriate format. After the conversion and basic image processing steps, the lungs area is separated from the background and other tissues. With the final form of the CT images, the system segments the Covid-19 affected regions and calculates some relevant metrics, such as the affected area to lung area ratio. Later on, authorized users can give structured feedback on the findings.

Image segmentation algorithms are used for both the lung segmentation and the infection segmentation. Image segmentation [45] is the method of dividing or segmenting the image into multiple regions based on the similarity or dissimilarity of the characteristics of those regions. Image segmentation is used then to extract the required objects from the background or other parts of the image. The various pixel characteristics like intensities, edges, curves, textures etc. are used as features to differentiate between the pixels for grouping the pixels with similar features and finally generate the segmented ROI.

Medical image segmentation is in general a very popular research area where various medical images (i.e. MRI, CT, X-ray, PET, etc.) are used as inputs to segment different organs/tissues (i.e. liver, kidney etc.) or abnormalities (i.e. tumors, lesions, fractures, etc.) from the images. Different ML, DL, TL, hybrid algorithms have been used for the medical image segmentation with high accuracy.

### Pre-processing

The images to be analyzed have to be pre-processed for consistency of formats, dimensions, etc. This pre-processing contains two main parts—(i) simple image processing, and (ii) lungs segmentation.

#### Image processing

Two main types of images are used in this study, namely PNG/JPG and DICOM. Most of the publicly available early Covid-19 CT datasets, and the datasets used in this study are in the PNG or JPG formats [46–50]. Therefore, the models are trained and tested with RGB and grayscale images. However, since most medical imaging systems use DICOM, and the developed system takes images directly from these systems via PACS, an intermediate step has been added to convert DICOM format images to PNG format. The ‘pydicom’ library [51] is used for this conversion process. Images in DICOM format are converted to PNG format by applying modality LUT and VOI LUT transformation [52, 53].

The pseudocode for pre-processing is shown in Algo 1. The input to the pre-processing is the lung image (i.e. X-rays/ CTs etc.) and the output is the pre-processed image. As mentioned earlier, the system can process PNG, JPG and DICOM files, but for DICOM files it converts the DCM images into PNG images. Hence at the beginning, the system checks if the image is in DICOM format and if it is then the system first applies modality LUT and then VOI LUT transformation and converts the DICOM image into a PNG image. In the next step, the image is normalized so that all pixel values are in the range 0 to 255. If the input image is PNG/JPG, then the conversion is bypassed and the image is directly normalized between 0 to 255. After the normalization, the lung regions are segmented from the image and extracted for both the right and left lung.

**Algorithm 1** Pre-processing: Image processing

**Require**: Input Image

 **if** image_type == DICOM **then**

  *Image* ← *apply*_*modality*_*lut*(*Image*)

  *Image* ← *apply*_*voi*_*lut*(*Image*)

 **end if**

 *Normalize Image between* 0 − 255

 *lung*_*mask* ← *LungSegmentation*(*Image*)

 *Image* ← *composite*(*Image*, *lung*_*mask*, *lung*_*mask*)   ▹ Separate the lung area

#### Lung segmentation

To achieve a more accurate Covid-19 affected area segmentation and to calculate the metrics about the lungs capacity such as the affected area to lungs area ratio, lungs segmentation and extraction processes are applied as the second part of pre-processing before the infected area segmentation. Throughout the study, the lungmask tool [54] and a similar implementation of the U-Net by [55, 56] are used to segment the lungs area. The lungmask’s pre-trained ‘U-Net(R231)’ model is used to segment the lungs directly from the DICOM images [54], and a model similar to Lee’s U-Net [56] model is retrained with both ‘Finding and Measuring Lungs in CT Data’ dataset [57] and ‘CNCB CT scan’ dataset [47] to segment the lungs area in the PNG/JPG images. With the masks obtained, other tissues are removed so that the tissue outside the lungs area is in the background of the image.

Algo 2 shows the steps of the lung segmentation process. A processed image from the previous step is used as input and the segmented lungs are produced as outputs. The processed image is resized into the dimension of (256, 256, 1). A basic thresholding with pixel score 190 is applied so that the pixels with values greater than 190 are replaced by 255. Then the image is fed into the pre-trained DNN for prediction. If the prediction is less than 0.5 then the pixel is not counted (i.e. assigned 0 as pixel value), otherwise 1 is assigned to the pixel. Then prediction markers are created from the threshold outputs and Sobel filtering is used to generate the elevation map for the prediction. Watershed thresholding is then used to enhance the edges and holes in the ROIs are filled in extracted regions. Finally, small unnecessary regions are removed with thresholding and the final ROIs are combined to get the lung masks for both the right and left lungs.

**Algorithm 2** Pre-processing: Lung Segmentation

**Require**: Image (i.e. Pre-processed Image)

 *Image* ← *resize*(*Image*, (256, 256, 1))

 *Image*[*Image* > 190]←255     ▹ Apply thresholding

 *model* ← *load*_*model*()

 *prediction* ← *model*.*predict*(*Image*)

 *prediction*[*prediction* < 0.5] ← 0    ▹ Apply threshold to prediction

 *prediction*[*prediction* > 0.5] ← 1

 *Create markers from prediction*

 *elevation*_*map* ← *sobel*(*prediction*)

 *regions* ← *watershed*(*elevation*_*map*, *markers*)

 *regions* ← *binary*_*fill*_*holes*()     ▹ Fill in found regions

 *Apply threshold to eliminate small regions*

 *Combine found regions*

 *mask* ← *resize*(*combined*_*image*, *original*_*image*_*size*)

### Infected area segmentation

The infected area from the lungs is segmented with three different DNN approaches—(i) Mask R-CNN [58], (ii) U-Net [55], and (iii) U-Net++ [59]. These DNN models are applied on the lungs individually. All the three methods are trained and tested with the lung area extracted from grayscale CT images generated by Algo 2. The segmented lungs are used as input images for the DNNs to segment them even further based on the infection regions. Based on the model chosen by the user from Mask R-CNN, U-Net and U-Net++, the DNN is trained with the lung segmented images and the predictions are generated as outputs containing the infection regions.

**Fig 2:**
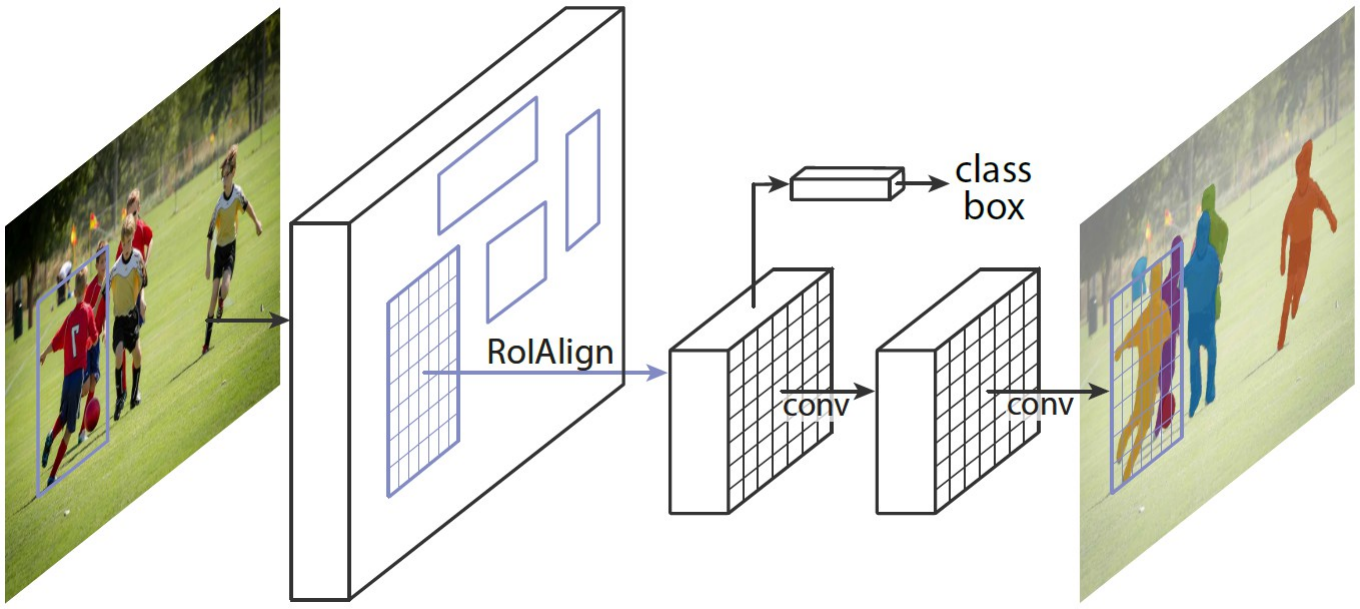
Mask R-CNN framework [58].

Mask R-CNN is a DNN for high performance instance segmentation and object classification [58]. Convolutional Neural Networks (CNN) are used on image data to optimize the pixel information for image processing and analysis. CNNs and it’s variations are very popular in image segmentation tasks and the basic CNN structure includes convolution layers, pooling layers and fully connected layers for image analysis. Convolution layers are used to generate feature maps from the input images using various filters and kernels, whereas the pooling layers generates summarized scores of different patches of the feature maps to downsample it. The fully connected layers are used to connect all neurons of one layer of the artificial neural network to every neuron of the other layer. Region-based CNN (R-CNN), a variation of CNN applied for image detection is used for detection of multiple regions from an image by applying CNNs on each ROI bounding boxes from the input image. Fast R-CNN is a variation of R-CNN that applies classification and bounding box regression on features extracted by ROI pooling. After combining learning from fast R-CNN and attention mechanism with Region Proposal Network (RPN), a Faster R-CNN model is developed that also enhances the computation speed of the DNN. The RPN generates more appropriate region proposals for object bounds. Mask R-CNN is an extension of the Faster R-CNN [60] that includes a branch for object masks prediction with the original object bounding box extraction. The Faster R-CNN has two stages. First, the RPN extracts objects and creates bounding boxes for them, and then a feature extraction is applied on objects within the bounding box for object classifications. Mask R-CNN also performs these stages. But at the second stage, it creates a binary mask for the objects in parallel to object classification. Although Mask R-CNN adds a little overhead to Faster R-CNN, it is more efficient with the advantage of having a very simple training and testing phase. The basic Mask R-CNN framework is shown in Fig 2.

The Mask R-CNN model for the proposed system is based on Arem Ter-Sarkisov’s implementation of Mask R-CNN, which introduces a more lightweight model [61, 62]. The problem is handled as a multiclass segmentation problem with ground-glass opacity, consolidation and background classes, and a binary segmentation problem with Covid-19 affected area and background classes in [61]. However, our study uses only a binary segmentation model by merging masks in both the introduced dataset and the CNCB CT scan dataset [47] which was used in the Ter-Sarkinov model [61].

**Fig 3:**
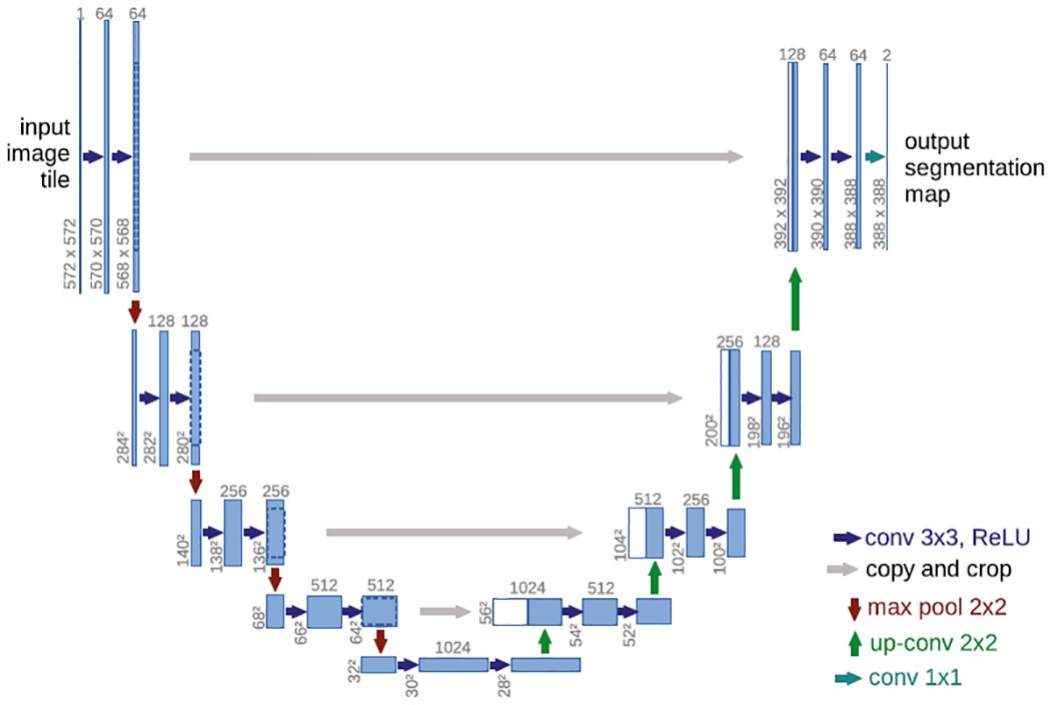
U-Net framework [55].

U-Net, on the other hand, is a fully connected convolutional network developed for more precise and faster biomedical image segmentation [55]. The framework includes an encoder and a decoder to classify each pixel and extract the features at each stage for semantic learning. The semantic segmentation model includes a contracting path and an expansive path. The contracting path contains convolutional layers with downsampling whereas the expansive path consists of convolutional layers and upsampling with 23 convolutional layers in total. Each level of each path extracts more dense features from the input image and combines them with the previous features. The contraction path has five levels and the expansive path includes four levels. Each level of the contraction path has two convolutional layers for feature extraction and one max pooling layer for reducing the image size. At every level, the depth gradually increases as the size of the image decreases and the network learns the semantic features. The expansive path works in the reverse direction. At each level, there is a transposed convolution with the regular convolution layer. Instead of maxpooling, it has upsampling process to increase the size of image while decreasing the depth. To incorporate the location information more appropriately, skip connections are used at each level of the expansive path by concatenating the output with the output of the same level of the contracting path. The structure of the network resembles a symmetric U shape. The U-Net framework is shown in Fig 3. The U-Net has been one of the most popular DNN models for medical image segmentation, and multiple variations of U-Net have also been developed to improve the performance even further.

**Fig 4:**
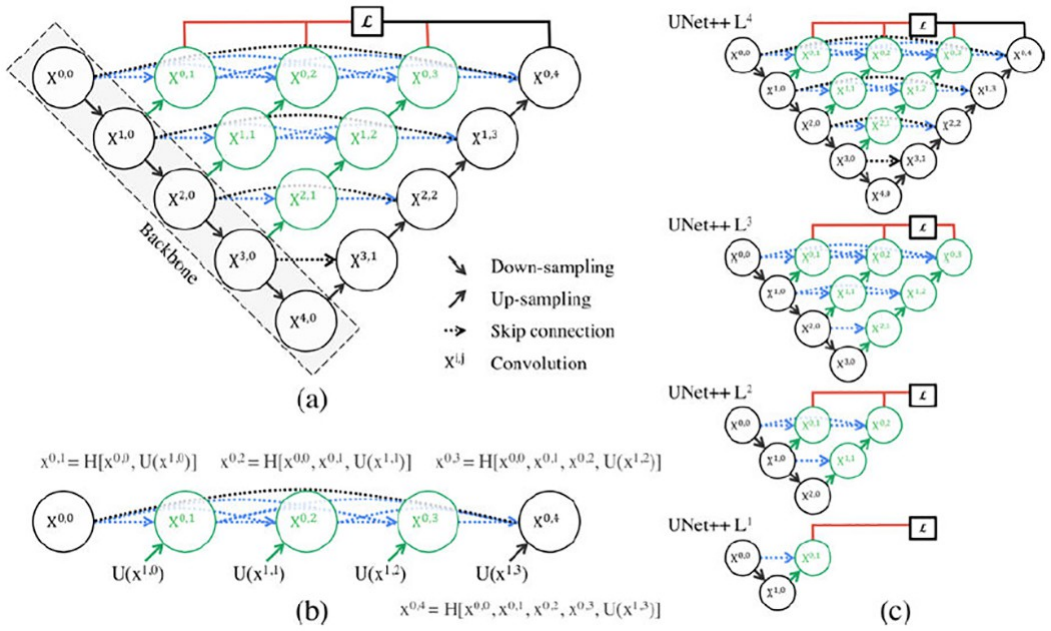
U-Net++ framework [59].

U-Net++ is an extension of the original U-Net framework [59]. U-Net++ is a nested U-Net that extends U-Net by adding modified skip pathways, skip connections and deep supervisions between the layers of contracting and expansion paths. Skip pathways reduce the semantic gaps between the encoder and decoder by directly connecting their feature maps. Dense skip connections are added based on the DenseNet [63] idea, and these skip connections accumulate the feature maps from all the intermediate nodes. Finally, a deep supervision step averages the branch outputs and selects one for faster and accurate segmentation. As the modified dense layer of U-Net++ adds more skip connections, it basically introduces more deep semantic features by reducing the semantic gap between the encoder and the decoder. To reduce the semantic gap, the skip connections add not only the features from the same level of the encoder, but all of other previous levels. This modification enables the DNN to incorporate the feature maps from different levels to create more specific feature maps for the image analysis. The deep supervision part of the model balances the performance and the computation time of the model. The deep supervision takes an average of the segmentation outputs of all layers and uses that as an additional input to the final calculation. The U-Net++ framework is shown in Fig 4. The basic U-Net and U-Net++ structures are implemented for our study following the frameworks mentioned in [55, 59] by using keras models [64].

To summarize, the major differences between U-Net and U-Net++ are—(i) U-Net++ reduces the semantic gaps between the feature maps of the encoder and the decoder path, (ii) U-Net++ improves the gradient flow with the dense skip connections, and (iii) U-Net++ improves the performance with deep supervision layer [65]. The differences can be seen in their structures from Figs 3 and 4 where the black parts in Fig 4 are from the original U-Net, the green parts are the added dense blocks, the green and blue arrows are the added skip connections, and the red parts are the added deep supervision. The added dense convolution blocks help to reduce the gaps between the same level of the encoder and decoder path. So, the feature maps generated at the same level are more similar in U-Net++ which simplifies the learning process. The skip connections connect the original U-Net backbone and the added dense blocks so that the outputs of convolution blocks of the same layers are combined and merged with the outputs of the lower level of the encoder path. This process helps to incorporate the feature maps with more semantic similarities. Finally, the deep supervision of U-Net++ allows the model to apply one of the two modes (i.e. accurate mode, fast mode). The accurate mode takes the average of all segmentation outputs and the fast mode chooses one of the segmentation outputs based on model pruning and speed gain. In general, U-Net++ provides a more optimized and accurate segmentation model compared to original U-Net.

The infection segmentation process is included in Algo 3. The system loads the model based on the choice of the user. Once one of the models from the Mask R-CNNs, U-Net, and U-Net++ is chosen, the prediction for the input image is generated and the confidence threshold is applied. For the mask R-CNN model, the system needs to combine the generated infection segmentation regions. The infection region is then combined with the original image for the visualization of the infection and the corresponding evaluation scores are computed.

**Algorithm 3** Lung Infection Segmentation

**Require**: Image (i.e. Segmented Lung Image)

 *model* ← *load*_*model*()

 *prediction* ← *model*.*predict*(*Image*)

 *Apply confidence threshold*

 **if** model.type == MaskRCNN **then**

  *Combine found regions*

 **end if**

 *mask* ← *prediction*.*mask*

 *Combine original image and the mask*

 *Save mask*, *combined*_*image*, *prediction scores*

### Post-processing

The infection segmentation process extracts the infectious parts from the lungs and then a post-processing method is applied to refine the results and calculate the ratio of the infection. Algo 4 shows the steps of the post-processing. The original images, lung masks and the Covid infection masks are used as inputs for the post-processing. As the lung masks have only the lung regions, the pixels with non-zero values are counted to calculate the lung area. The same method is applied to calculate the infected area from the infection masks. Then the ratio of the infection is computed from these two values and shown as part of the results in the UI.

**Algorithm 4** Post-processing

**Require**: Images, LungMasks, CovidMasks

 *results* ← []

 *total*_*lung*_*size* ← 0

 *total*_*infected*_*area*_*size* ← 0

 *total*_*ratio* ← 0

 **for** *image in Images* **do**

  *lung*_*size* ← *count*_*nonzero*(*LungMask*)

  *total*_*lung*_*size* + = *lung*_*size*

  *infected*_*area*_*size* ← *count*_*nonzero*(*CovidMask*)

  *total*_*infected*_*area*_*size* + = *infected*_*area*_*size*

  **if** *lung*_*size* > 0 **then**

   *covid*_*ratio* ← *infected*_*area*_*size* / *lung*_*size*

  **end if**

  *results*.*append*((*lung*_*size*, *infected*_*area*_*size*, *covid*_*ratio*))

  *total*_*ratio* ← *total*_*infected*_*area*_*size* / *total*_*lung*_*size*

**end for**

### UI—Web application

The web application introduced in this study has been designed as a complete framework for clinical usage, and for further research usage. The system is designed as a Flask web application. On the client side, pages are generated with the Jinja template engine. PACS and Feedback pages have additional functionalities implemented with HTML and Javascript. On the PACS page, asynchronous queries are implemented with jQuery. A modified version of VGG Image Annotator [34] is used for gathering user feedback.

**Fig 5:**
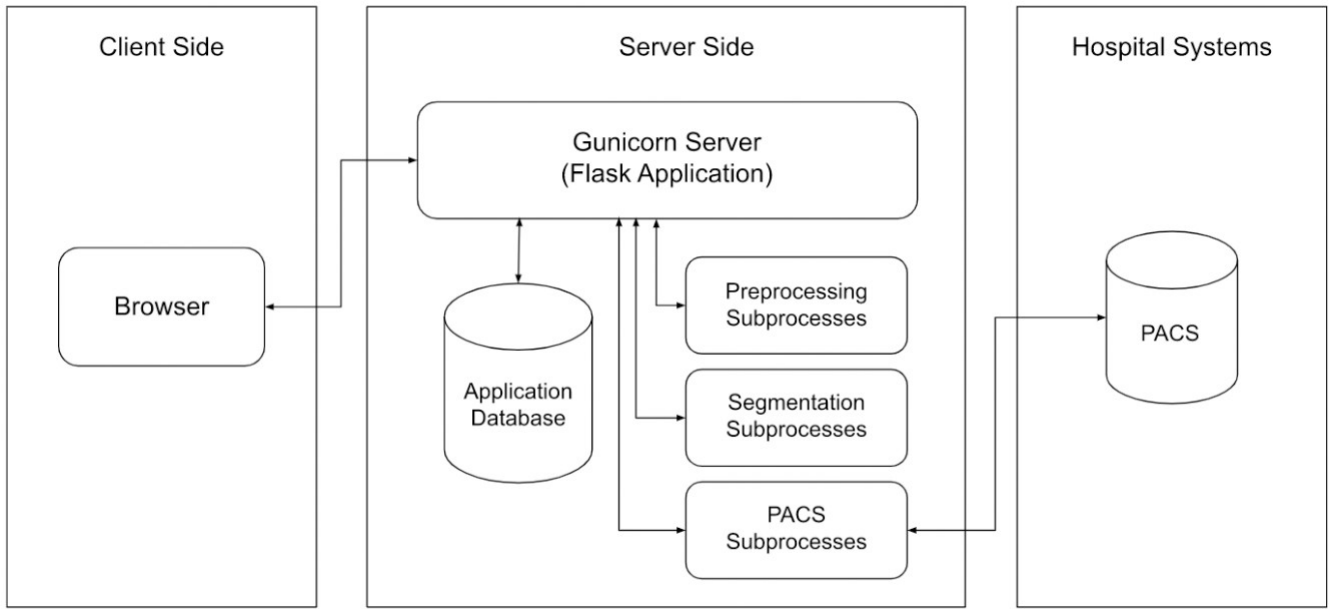
Web application architecture.

The system architecture is depicted in Fig 5. The main component of the server side is the Gunicorn WSGI server which is used for running the main Flask application. Additionally, there is a PostgreSQL database to store user information and evaluation logs. Session-based authentication is used for For access control. Time-consuming parts of the system such as segmentation and PACS communication are designed in a modular structure as subprocesses. With this structure, segmentation methods are hot-swappable and new methods can be easily added even if they are written in another programming language. Communication with the PACS was established with C-ECHO, C-FIND, and C-MOVE requests. With the C-ECHO [5] request, the system checks that the PACS is accessible and active. The C-FIND message allows the users to search with the parameters entered. In the last step, the selected DICOM images are requested over PACS with C-MOVE.

**Fig 6:**
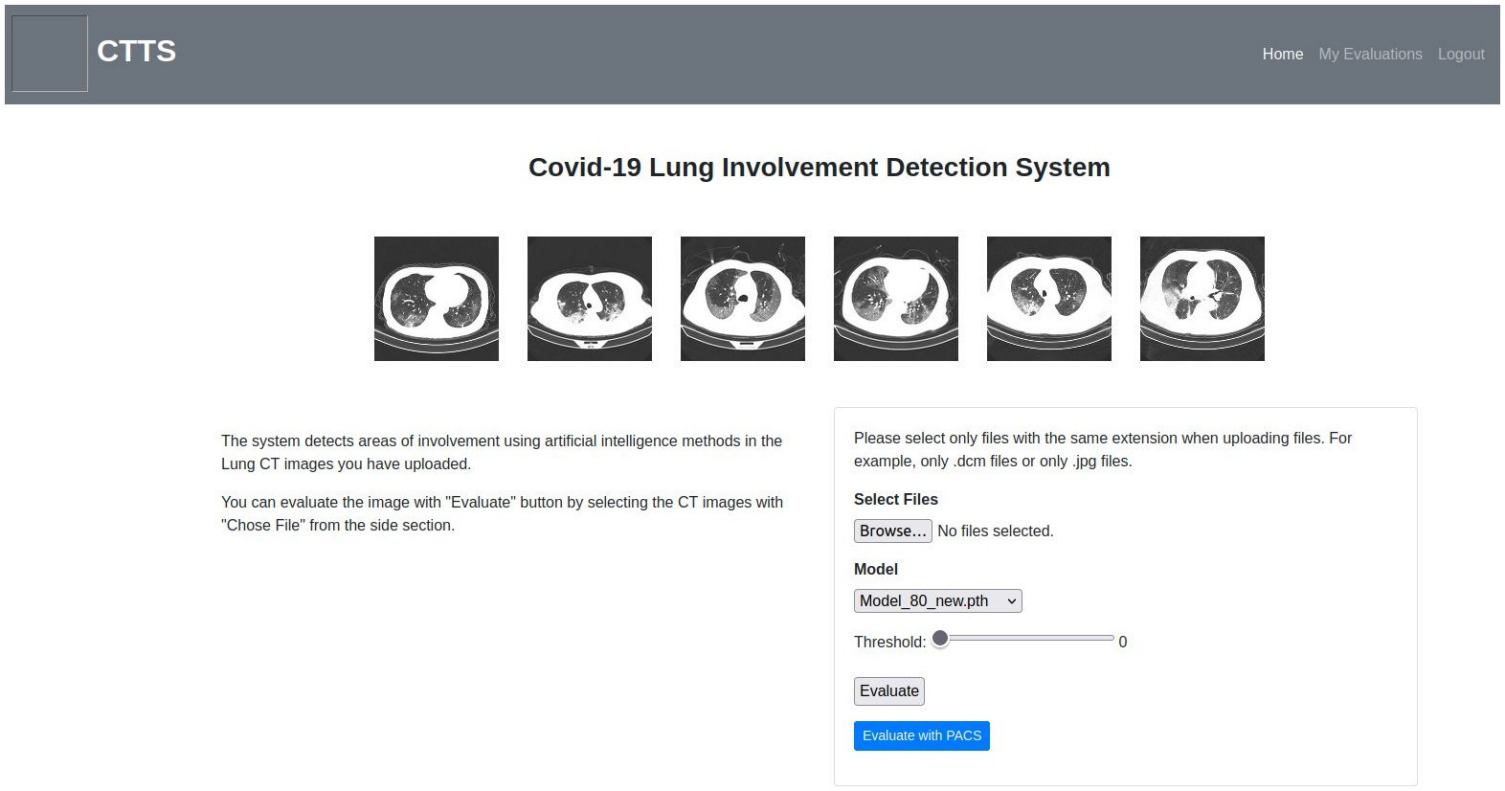
Home page.

**Fig 7:**
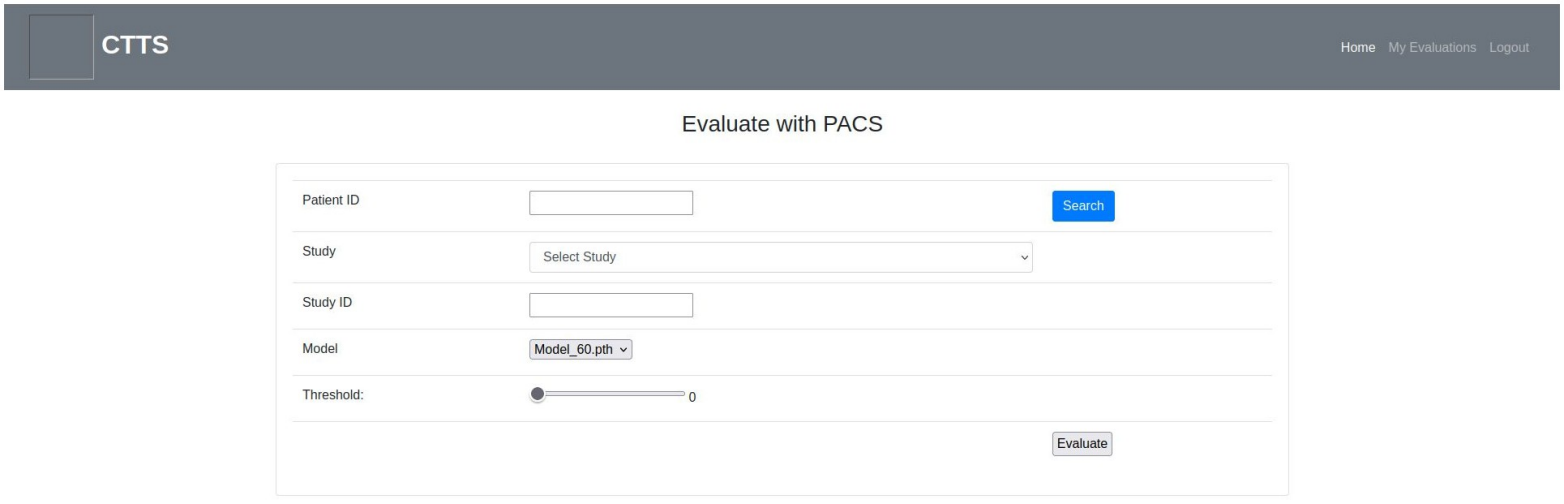
Evaluate with PACS page.

**Fig 8:**
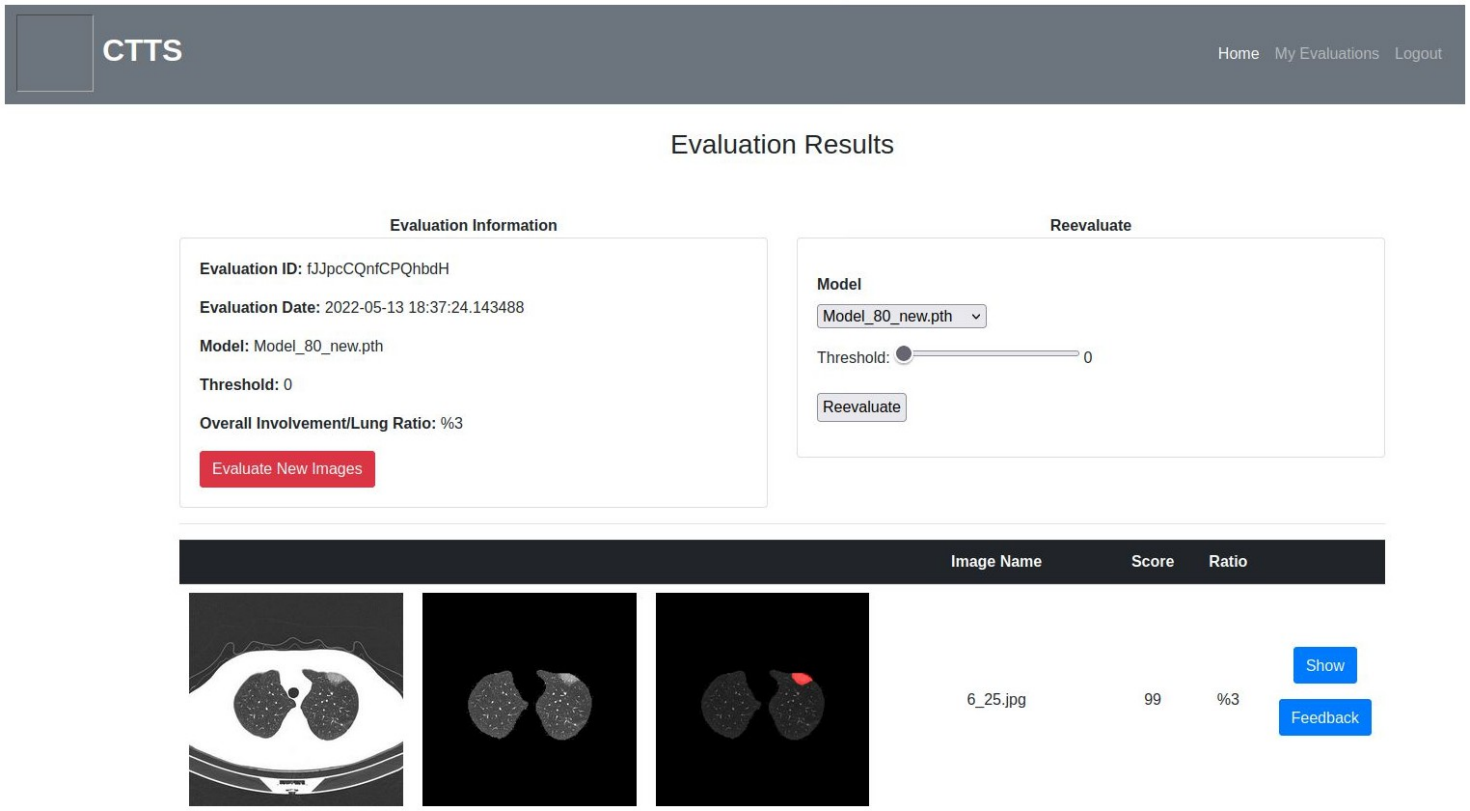
Results page.

**Fig 9:**
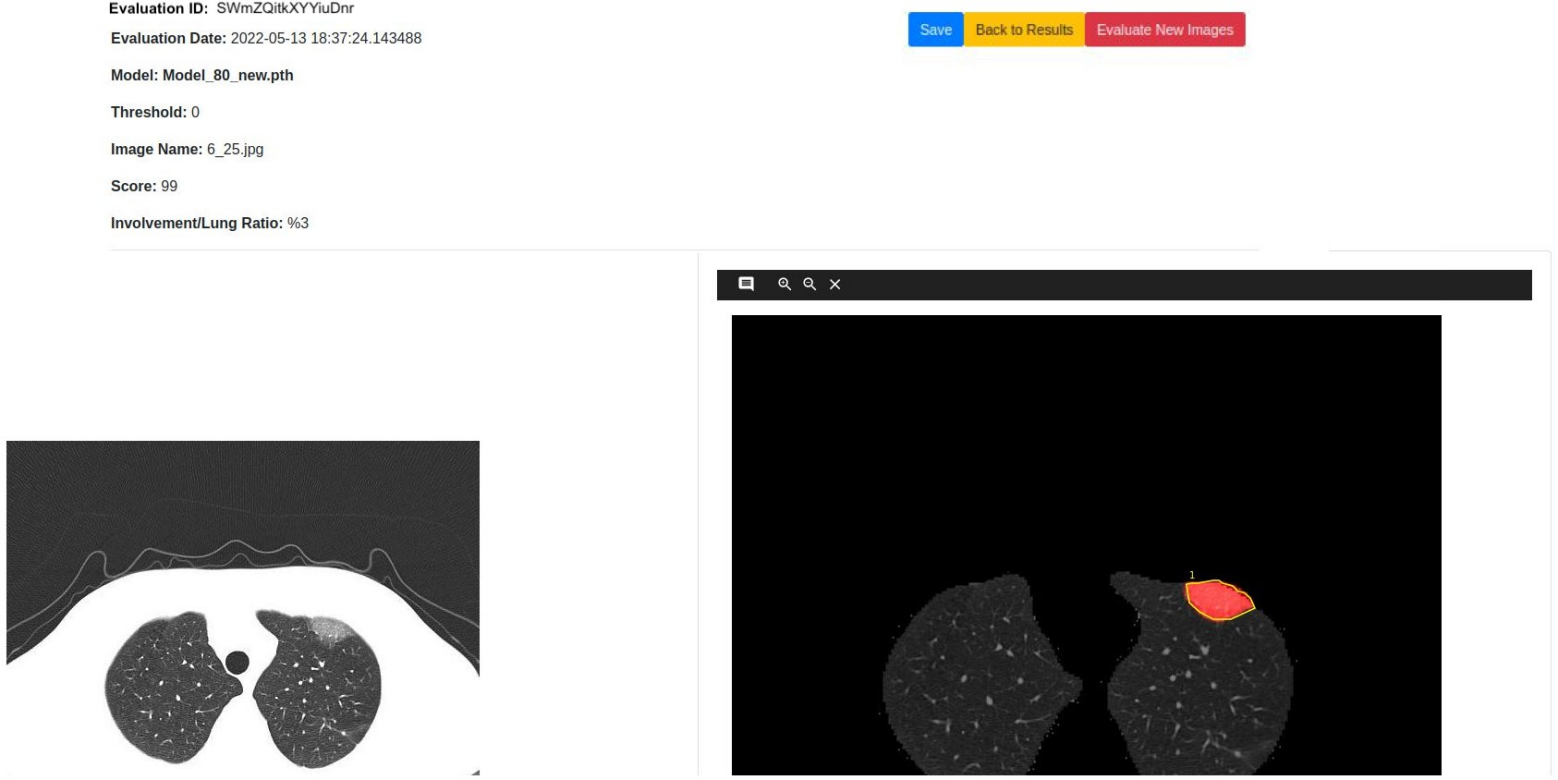
Part of the feedback page.

There are screenshots from the user interface in Figs 6–9. There are two ways to evaluate images in the system. One option is by uploading PNG, JPG, or DICOM files to be evaluated with the file upload option shown in Fig 6. The second option is evaluating the images selected via PACS which can be accessed via the ‘Evaluate with PACS’ button. Besides the file upload option, there are two other parameters indicated in Fig 6, specifying the model to be used and deciding on the associated threshold value. Users can select between Mask R-CNN, U-Net, and U-Net++ models with different training parameters. The threshold parameter gives users the opportunity to filter out findings that have inference accuracy lower than the specified threshold.

Using the page shown in Fig 7, images can be evaluated through PACS rather than uploading manually. Users can browse through and select previous CT scans of patients based on Patient ID. Scan date, body part examined, and modality information are available to the users. There are also models to be used and associated threshold options shown in Fig 7. When users interact with PACS, such as searching patient ID or selecting images for evaluation, the application communicates with PACS via ‘DICOM Query/Retrieve Service’ [66] using pynetdicom library [67]. Once the CT scans are selected, the evaluation starts with the previously mentioned pre-processing step.

In the results page, which is shown in Fig 8, there are three sections. The upper left section contains evaluation ID and date, the used parameters, and the total Covid-19 affected area to lungs area ratio. The upper right section is used to re-evaluate the same images with different thresholds and models without re-uploading. In the lower section, there is a table with the following columns—original image thumbnail, lungs image thumbnail, the thumbnail of the image with the found areas, image name, score, affected area-lungs area ratio, and two buttons which are used as shortcuts for the feedback and comparison pages.

**Fig 10:**
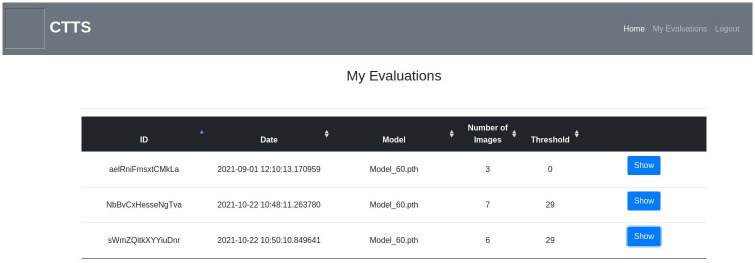
My evaluations page.

Users can also compare the original image and the masked image side-by-side via the show button. Users can annotate the masked image and give feedback about the evaluation on the feedback page shown in Fig 9. The feedback page is based on the simplified version of VGG Image Annotator (VIA) [68]. The annotated feedback is intended to be used for tune up to further improve the models. On ‘My Evaluations’ page (see Fig 10), each user can see their previous evaluations and brief information about the evaluation such as ID, date, model, number of images and threshold.

### Ethics

No participant consent is needed because we have used publicly available data. The data has been downloaded from the public site: http://ncov-ai.big.ac.cn/download.

## Experimental results

### Datasets

Our models used 902 chest medical images, combining the CNCB-NGDC [47, 69] dataset with some augmented images to increase the size of the dataset. The CNCB-NGDC dataset was created from images provided by China Consortium of Chest CT Image Investigation (CC-CCII). We used the Lesion segmentation dataset of the CT slice images from datasets containing 750 CT slices taken from 150 COVID-19 patients. Each CT slice of the dataset is segmented and annotated into four components—background, lungs field, ground-glass opacity (GGO), and consolidation (CL). Additionally, 152 images were generated by augmentation of the other images already in the dataset, and added to enrich the dataset. The latter images were checked manually for validity and diversity and were marked with annotations of the infection regions. The total dataset with all 902 images were used for our experiments with Mask R-CNNs, U-Net and U-Net++ models. 629 images were used for training the deep neural networks, 158 images were used as the validation data and the rest (i.e., 115 images) were used as the test data.

### Experimental setup

The image segmentation models were applied for two types of segmentation—(i) lung segmentation, and (ii) infection segmentation. The hyperparameters used for the lung segmentation and infection segmentation tasks were similar. Both tasks used model optimizer, learning rate, number of epochs, batch size, dropout, activation function, loss function etc. [70–72]. The ‘optimizer’ of a DNN is an algorithm or method that modifies the values of attributes of the DNN to minimize the loss of the DL model [73]. Stochastic Gradient Descent, Adagrad, Adam, Adadelta are some examples of common optimizers used in DL models [74, 75]. Each of them varies based on their convergence rate, computation cost, tuning etc. and each DL task uses an optimizer most suitable for the application. The ‘activation function’ is the function that decides if a neuron of the neural network should be activated or not [76]. Depending on the importance of the output of each neuron, the activation function adds non-linearity to the network and decides whether a neuron should be activated to contribute it’s output generated from the connected neurons of the previous layer to the network. Rectified linear unit (ReLU), Sigmoid, Softmax are some commonly used activation functions [77]. The ‘dropout’ is a regularization process to avoid overfitting the DNN [78, 79]. It randomly drops out neurons/nodes from a fully connected DNN layers (i.e. input layer and hidden layers) based on a given dropout probability in every iteration. The ‘learning rate’ of a DNN represents the amount of changes needed at every weight update of the model based on the estimated error [80]. The convergence of the model for an optimal solution depends on the learning rate. The convergence can be too slow if the learning rate is low, and the training can be unstable with high learning rate and both can lead to never reaching to an optimal solution. The ‘number of epochs’ is a DL model is the number of times the model passes through the complete dataset for training the model. The ‘batch size’ is different from the epochs [81]. Instead of passing the whole dataset at once, the dataset is generally divided into sets of data to train the DL model. The number of data in one such set or batch is called the batch size. The number of epochs and batch sizes are decided based on the dataset, dataset size and the diversity of the data. The ‘loss function’ measures the dissimilarities between the predicted output and the ground truth [82]. The goal for any DNN is to minimize the loss by applying various types of regression (i.e. mean squared error, mean absolute error, etc.) and classification (i.e. binary cross entropy, categorical cross entropy, etc.) loss functions or generating hybrid or novel loss functions.

The model used for lung segmentation has similarities with U-Net as it has a downsampling path and an upsampling path. There is a total of 331,137 trainable parameters. Adam and Binary Cross Entropy has been used as the optimizer and the loss function. Table 1 shows the model details of the lung segmentation FCNN.

**Table 1:** Lung segmentation model.

Layer	Type	Output Shape	Kernel Size
0	Input Layer	(256, 256, 1)	-
1	Convolution (ReLU)	(256, 256, 32)	3x3
2	Max Pooling	(128, 128, 32)	2x2
3	Convolution (ReLU)	(128, 128, 64)	3x3
4	Max Pooling	(64, 64, 64)	2x2
5	Convolution (ReLU)	(64, 64, 128)	3x3
6	Max Pooling	(32, 32, 128	2x2
7	Dense (ReLU)	(32, 32, 128)	-
8	Up-Sampling	(64, 64, 128)	2x2
9	Convolution (Sigmoid)	(64, 64, 128)	3x3
10	Up-Sampling	(128, 128, 128)	2x2
11	Convolution (Sigmoid)	(128, 128, 64)	3x3
12	Up-Sampling	(256, 256, 64)	2x2
13	Convolution (Sigmoid)	(256, 256, 1)	3x3

The infection segmentation task used Mask R-CNN, U-Net and U-Net++ for the infectious region segmentation. The Mask R-CNN algorithm was applied with three different versions by varying the number of epochs or iterations. Mask R-CNN 40, Mask R-CNN 60 and Mask R-CNN 80 are Mask R-CNN algorithms with 40, 60 and 80 epochs, respectively. Each of these versions was applied with ten different Intersection over Union (IoU) thresholds (i.e. 0.50, 0.55, 0.60, 0.65, 0.70, 0.75, 0.80, 0.85, 0.90, and 0.95) to generate 30 Mask R-CNN models in total. The Mask R-CNN models used pre-trained ResNet backbones for the DL model. ResNet18 ResNet34, and ResNet50 models were applied as the backbone; ResNet50 is the default model. The model weights, anchor generator, optimizer state, etc. were assigned from the pre-trained models. The training phase used learning rate 0.00001 for epochs 40, 60 and 80, respectively. The test phase used similar parameters with 0.05 confidence score threshold and 0.5 mask threshold. The test phase also used the aforementioned IoU thresholds for the precision scores. The ground truth masks included infections labeled as Ground Glass Opacity (GGO), Consolidation (C), or both.

The U-Net used the 2D image slices to train and validate the model with 80% data and test it with 20% data. The training dataset was used for data augmentation by flipping the images and the ground truth to the left and to the right individually. The flipped images were added to the training dataset and the U-Net model was trained with a batch size of 8 for 100 epochs. The model used Adam optimizer with learning rate 0.001. The kernel size for all layers of the U-Net was 3x3, stride was 2x2, and the filter sizes were 64, 128, 256, 512, 512 and 512, 256, 128, 64 for the contraction and expansion layers, respectively. Each contraction layer had a 2x2 MaxPooling layer and Batch normalization layer with momentum 0.8. A dropout of 10% was applied at the end of each contraction layer. The U-Net model used a hybrid loss function combining the binary cross entropy and dice loss. Each loss function contributed to the total hybrid loss calculation with 0.5 weight. All convolution layers used the RelU activation function, whereas the final convolution block used the Sigmoid activation function to generate the output. The U-Net for the infection segmentation task used 22,716,609 trainable parameters in total.

The U-Net++ model applied a very similar set of functions and hyperparameters. The structure for the U-Net++ includes one contraction layer and four expansion levels with 1, 2, 3, and 4 expansion layers to merge the results of the contraction and expansion layers, respectively. The filter sizes for the expansion levels were 64 (level 1); 128, 64 (level 2); 256, 128, 64 (level 3); and 256, 256, 128, 64 (level 4). The loss function, activation functions, stride size, contraction layer filter sizes, Batch normalization, MaxPooling and other parameters for U-Net++ were similar to those of the aforementioned U-Net model setup. The batch size and number of epochs for U-Net++ training were 8 and 100 respectively. U-Net++ also used the hybrid of binary cross entropy and dice loss as the loss function. After calculating the binary cross entropy and dice loss separately, each of them was added with 0.5 weight to generate the total loss. The activation function used for all convolution layers except the final block was RelU and the final convolution block applied Sigmoid function. The learning rate of the model for the Adam optimizer was 0.001, the momentum of the Batch normalization is 0.8, and the dropout after each contraction layer was 10%. The Maxpooling layer at each contraction layer had a pool size 2x2. The kernel size and strides in all layers were 3x3 and 2x2 respectively. The U-Net++ for the infection segmentation task used 22,496,961 trainable parameters in total. Tables 2 and 3 show the U-Net and U-Net++ model structures for the infection segmentation.

**Table 2:** Infection segmentation model: U-Net.

Layer	Type	Output Shape	Kernel Size
0	Input Layer	(128, 128, 1)	-
	Convolution (ReLU)	(128, 128, 64)	3x3
	Convolution (ReLU)	(128, 128, 64)	3x3
1	Max Pooling	(64, 64, 64)	2x2
	Batch Normalization	(64, 64, 64)	-
	Dropout	(64, 64, 64)	-
2	Convolution (ReLU)	(64, 64, 128)	3x3
	Convolution (ReLU)	(64, 64, 128)	3x3
	Max Pooling	(32, 32, 128)	2x2
	Batch Normalization	(32, 32, 128)	-
	Dropout	(32, 32, 128)	-
3	Convolution (ReLU)	(32, 32, 256)	3x3
	Convolution (ReLU)	(32, 32, 256)	3x3
	Max Pooling	(16, 16, 256)	2x2
	Batch Normalization	(16, 16, 256)	-
	Dropout	(16, 16, 256)	-
4	Convolution (ReLU)	(16, 16, 512)	3x3
	Convolution (ReLU)	(16, 16, 512)	3x3
	Max Pooling	(8, 8, 512)	2x2
	Batch Normalization	(8, 8, 512)	-
	Dropout	(8, 8, 512)	-
5	Convolution (ReLU)	(8, 8, 512)	3x3
	Convolution (ReLU)	(8, 8, 512)	3x3
6	Transposed Convolution	(16, 16, 512)	3x3
	Concatanate (Layer 4 Convolution)	(16, 16, 1024)	-
	Dropout	(16, 16, 1024)	-
	Convolution (ReLU)	(16, 16, 512)	3x3
	Convolution (ReLU)	(16, 16, 512)	3x3
7	Transposed Convolution	(32, 32, 256)	3x3
	Concatanate (Layer 3 Convolution)	(32, 32, 512)	-
	Dropout	(32, 32, 512)	-
	Convolution (ReLU)	(32, 32, 256)	3x3
	Convolution (ReLU)	(32, 32, 256)	3x3
8	Transposed Convolution	(64, 64, 128)	3x3
	Concatanate (Layer 2 Convolution)	(64, 64, 256)	-
	Dropout	(64, 64, 256)	-
	Convolution (ReLU)	(64, 64, 128)	3x3
	Convolution (ReLU)	(64, 64, 128)	3x3
9	Transposed Convolution	(128, 128, 64)	3x3
	Concatanate (Layer 1 Convolution)	(128, 128, 128)	-
	Dropout	(128, 128, 128)	-
	Convolution (ReLU)	(128, 128, 64)	3x3
	Convolution (ReLU)	(128, 128, 64)	3x3
10	Convolution (Sigmoid)	(128, 128, 1)	3x3

**Table 3:** Infection segmentation model: U-Net++.

Layer	Type	Output Shape	Kernel Size
0	Input Layer	(128, 128, 1)	-
1	Convolution (ReLU)(d1)	(128, 128, 64)	3x3
	Convolution (ReLU)(d1)	(128, 128, 64)	3x3
	Max Pooling(p1)	(64, 64, 64)	2x2
	Batch Normalization(p1)	(64, 64, 64)	-
	Dropout(p1)	(64, 64, 64)	-
2	Convolution (ReLU)(d2)	(64, 64, 128)	3x3
	Convolution (ReLU)(d2)	(64, 64, 128)	3x3
	Max Pooling(p2)	(32, 32, 128)	2x2
	Batch Normalization(p2)	(32, 32, 128)	-
	Dropout(p2)	(32, 32, 128)	-
3	Convolution (ReLU)(d3)	(32, 32, 256)	3x3
	Convolution (ReLU)(d3)	(32, 32, 256)	3x3
	Max Pooling(p3)	(16, 16, 256)	2x2
	Batch Normalization(p3)	(16, 16, 256)	-
	Dropout(p3)	(16, 16, 256)	-
4	Convolution (ReLU)(d4)	(16, 16, 512)	3x3
	Convolution (ReLU)(d4)	(16, 16, 512)	3x3
	Max Pooling(p3)	(8, 8, 512)	2x2
	Batch Normalization(p3)	(8, 8, 512)	-
	Dropout(p3)	(8, 8, 512)	-
5	Convolution (ReLU)(d4)	(8, 8, 512)	3x3
	Convolution (ReLU)(d4)	(8, 8, 512)	3x3
6	Transposed Convolution(u1)	(16, 16, 512)	3x3
	Concatanate[u1,d4](u1)	(16, 16, 1024)	-
	Dropout(u1)	(16, 16, 1024)	-
	Convolution (ReLU)(c1)	(16, 16, 512)	3x3
	Convolution (ReLU)(c1)	(16, 16, 512)	3x3
7	Transposed Convolution(u2)	(32, 32, 256)	3x3
	Concatanate[u2,d3](u2)	(32, 32, 512)	-
	Dropout(u2)	(32, 32, 512)	-
	Convolution (ReLU)(c2)	(32, 32, 256)	3x3
	Convolution (ReLU)(c2)	(32, 32, 256)	3x3
8	Transposed Convolution(u3)	(64, 64, 128)	3x3
	Concatanate[u3,d2](u3)	(64, 64, 256)	-
	Dropout(u3)	(64, 64, 256)	-
	Convolution (ReLU)(c3)	(64, 64, 128)	3x3
	Convolution (ReLU)(c3)	(64, 64, 128)	3x3
9	Transposed Convolution(u4)	(128, 128, 64)	3x3
	Concatanate[u4,d1](u4)	(128, 128, 128)	-
	Dropout(u4)	(128, 128, 128)	-
	Convolution (ReLU)(c4)	(128, 128, 64)	3x3
	Convolution (ReLU)(c4)	(128, 128, 64)	3x3
10	Convolution (Sigmoid)	(128, 128, 1)	1x1

### The results

The experiments on the dataset with Mask R-CNN, U-Net and U-Net++ using various parameters and setups showed some interesting characteristics of the infection detection and segmentation process. Accuracy, loss, precision, Jaccard score and Dice score were used as performance metrics in the experiments [83]. Table 4 shows performance comparisons between the average precision (AP) of three Mask R-CNN models (i.e., Mask R-CNN 40, Mask R-CNN 60, and Mask R-CNN 80) with IoU thresholds varying from 0.50 to 0.95 and their mean average precision (mAP). Precision represents the fraction of the prediction relevant to the ground truth (i.e., the purity of the prediction of true positives compared to the ground truth).

**Table 4:** Performance comparison between the Mask R-CNN segmentation models.

Model	mAP	AP (0.50)	AP (0.55)	AP (0.60)	AP (0.65)	AP (0.70)	AP (0.75)	AP (0.80)	AP (0.85)	AP (0.90)	AP (0.95)
Mask R-CNN 40	0.1427	0.3006	0.2672	0.2304	0.1851	0.1274	0.0884	0.0710	0.0530	0.0522	0.0522
Mask R-CNN 60	0.2193	0.3791	0.3555	0.3091	0.2549	0.2162	0.1673	0.1459	0.1217	0.1217	0.1217
Mask R-CNN 80	0.2352	0.4112	0.3864	0.3191	0.2757	0.2368	0.1711	0.1558	0.1348	0.1304	0.1304

The precision scores for all the 30 Mask R-CNN models with different epochs and varying IoU thresholds show how the models performance changed over the thresholds and the epochs. As the IoU thresholds define the threshold score to determine whether or not to return the prediction output generated by the model, the precision generally decreases with the increment of the threshold score. The precision scores for Mask R-CNN 40, Mask R-CNN 60, Mask R-CNN 80 followed the same pattern. The precision scores for lower thresholds (i.e., 0.50, 0.55, etc.) achieved high precision values for predictions of the segmentation model. The average precision scores for these three models showed that the Mask R-CNN 40 performance was considerably lower than that of the other two models whereas Mask R-CNN 60 and Mask R-CNN 80 had similar performances. For last few thresholds, the precision scores got saturated for all the models. Although Mask R-CNN 80 models outperformed the other two, Mask R-CNN 60 had around 2% performance difference compared to Mask R-CNN 80. The Mask R-CNN 40 models sometimes were able to find more pixels from the infection regions than the other two models, but it also detected more false positives than the other models while segmenting the infection regions from the images. In some cases, Mask R-CNN 80 models were not able to detect the complete infection region. They detected fewer false positive pixels compared to Mask R-CNN 40 and Mask R-CNN 60 models.

The U-Net and U-Net++ models were applied on the same datasets. Table 5 reports the accuracy and loss for U-Net and U-Net++ segmentation performance on the test data. Accuracy refers to the percentage of correctly classified pixels, and loss represents the difference between the prediction level and the ground truth. The U-Net and U-Net++ models achieved very high segmentation accuracy scores (i.e., more than 98%) with small amounts of losses. The original U-Net performed slightly better in terms of accuracy, but the U-Net model loss score was significantly lower than the U-Net++ loss score (i.e., about 12%). As the U-Net++ model is an advanced variation of U-Net with more layers and connections. The U-Net++ models generally work better with larger datasets. Due to the availability of limited amount of Covid-19 annotated datasets, the U-Net++ model loss score was higher. On the other hand, the basic U-Net model with fewer layers and connections than the U-Net++ model was able to more precisely segment the infection region.

**Table 5:** Performance comparison between U-Net and U-Net++ models.

Model	Accuracy	Loss
U-Net	0.9881	0.1361
U-Net++	0.9816	0.2523

**Fig 11:**
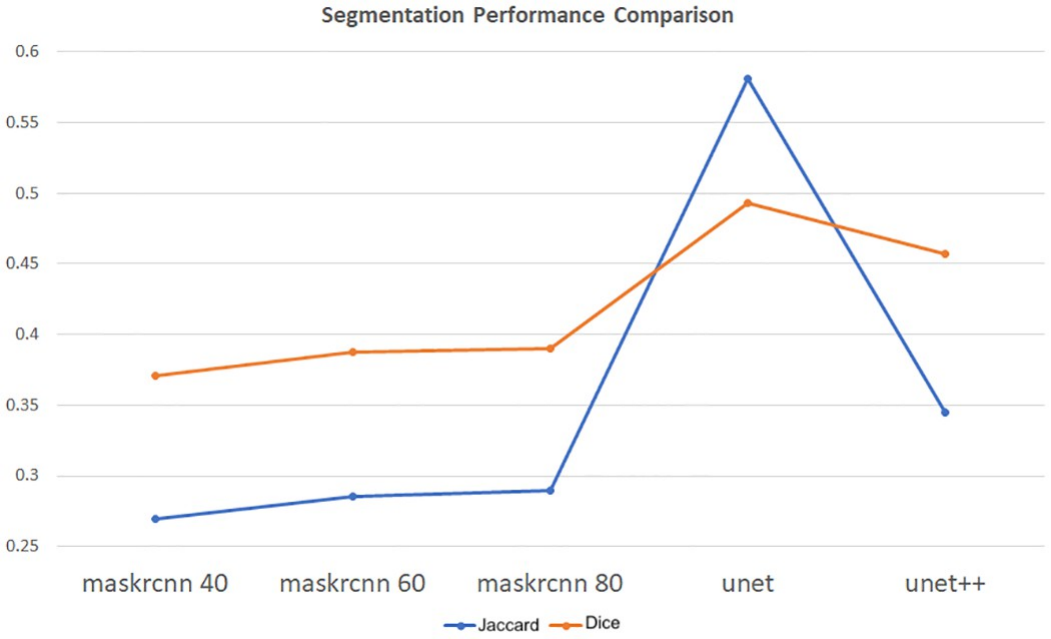
Performance comparison graph.

Table 6 reports the comparison between all these models (i.e., Mask R-CNN 40, Mask R-CNN 60, Mask R-CNN 80, U-Net, and U-Net++) with their Jaccard similarity score and Dice similarity score. Jaccard similarity score represents the fraction of the overlapping area between the segmentation and the ground truth with the union of both areas. Dice similarity score is a similar performance metric that refers to pixel-wise similarity between the ground truth and the segmented area which is the fraction of the overlap between them and the total number of pixels in both of them. Fig 11 shows the comparison of Dice similarity scores and Jaccard similarity scores of all DNN models related to the proposed system.

**Table 6:** Comparison between the segmentation models.

Model	Jaccard	Dice
Mask R-CNN 40	0.2692	0.3708
Mask R-CNN 60	0.2857	0.3875
Mask R-CNN 80	0.2894	0.3904
U-Net	0.5814	0.4927
U-Net++	0.3451	0.4574

**Fig 12:**
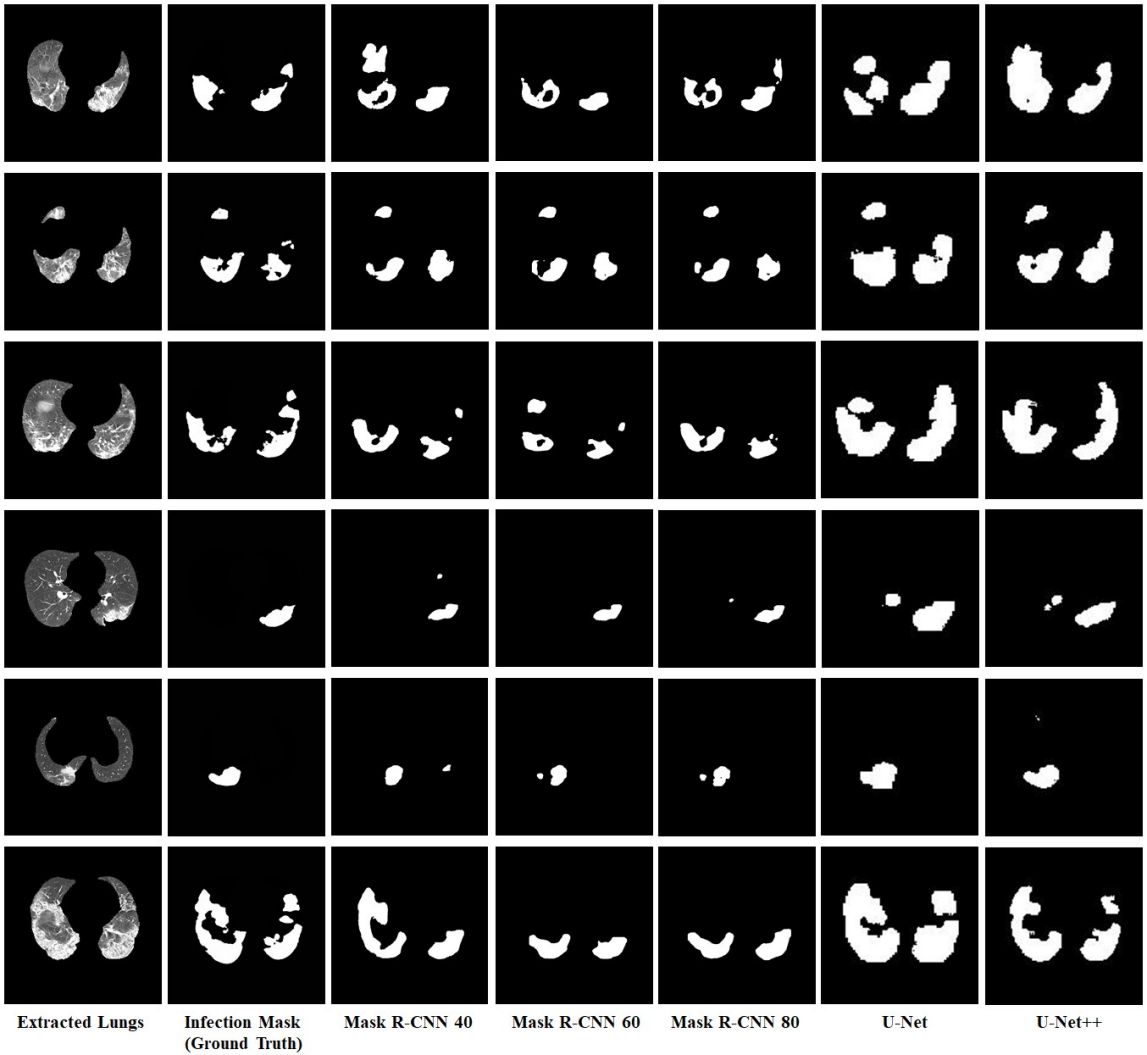
Sample segmentation outputs for all models.

The Jaccard and Dice similarity scores for all the models showed similar pattern as the individual Mask R-CNN model scores and U-Net models scores. The Mask R-CNN 80 models achieved high Dice and Jaccard scores, whereas the Mask R-CNN 60 models provided second best Mask R-CNN scores. The basic U-Net model Dice and Jaccard scores were at least 4% to 23% higher than the U-Net++ model. The difference between the Jaccard similarity scores of the best Mask R-CNN model (Mask R-CNN 80) and the U-Net model was around 10%, and the difference between the dice similarity scores was around 30%. These noteworthy differences between Mask R-CNN and U-Net models were also noticeable in the segmented output images. Fig 12 shows some sample segmentation output for all models with the extracted lungs image and infection ground truth.

The sample segmented images showed that Mask R-CNN models generated more precise segmentations compared to U-Net models. Mask R-CNN 80 models predicted fewer pixels for the segmentations than Mask R-CNN 60 or Mask R-CNN 40 models, but it was also able to segment most pixels from the infection regions similar to the ground truth images. The segmentation performance scores and the segmented images from Mask R-CNN clearly showed that the more trained models (i.e. higher number of epochs) performed better; as the models were trained for more epochs they were able to refine their detection and segmentation predictions. The performance scores showed that U-Net models performed better than Mask R-CNNs in segmenting the infection regions. The segmentation output images clarified the reasons underlying the performance. Both U-Net and U-Net++ models included wider regions as the segmentation output compared to the ground truth and Mask R-CNN output images. But the ground truth region pixels were almost always subsets of the segmented region pixels of U-Net and U-Net++ models, which contributed to the high Dice similarity scores and Jaccard similarity scores. The basic U-Net model outperformed all the other models by a high percentage and was able to capture most parts from the infectious regions of the chest CT images.

## Conclusion

AI-based automated Covid detection and diagnosis systems from medical chest images like chest X-rays or CTs can help medical professionals to quickly diagnose and help their patients. These systems can help patients by using non-invasive imaging for disease detection. As the tasks are based on medical images, high accuracy of the detection and precise infection segmentation are major priorities for such systems. Most recent research efforts have applied various deep learning models to address these issues and to improve the accuracy and precision as much as possible. In this paper, we propose a complete framework that provides a web application to incorporate the complete procedure of processing patient data and chest images, applying three different types of DL models (i.e., Mask R-CNN, U-Net, and U-Net++) with multiple variations, detecting Covid patients from the images, segmenting the infection in the lungs, marking the infection regions in CT images, and providing the ratio of the infectious area. This web application can also be used by medical professional to provide feedback on our segmentation and annotation output. The feedback can be used to tune up and refine the performance our system.

Experiments on three variations of Mask R-CNN (i.e., Mask R-CNN 40, Mask R-CNN 60, Mask R-CNN 80), U-Net, and U-Net++ models with two datasets showed impressive performances in Covid-19 region detection and segmentation from CT images. The U-Net being one of the most popular model for medical image analysis, outperformed all other segmentation models and showed comparable performance scores with more than 98% accuracy, and around 0.5 Dice similarity and Jaccard similarity scores with only 13% test loss. The other models performed well and the segmentation image outputs provided some precise infection region detection in most cases. Although our proposed novel approach of a complete framework for Covid-19 detection, segmentation and feedback submission to help healthcare professionals showed promising results, the performance of the infection detection process should be improved further to help healthcare professionals in better diagnosing Covid-19 patients and also expanding the same methodology to cover other types of diseases. We are currently working on improving the system to increase its acceptability and applicability. We want to refine the performance of the system by adding more DL and TL models (including hybrid and ensemble models). We also want to apply various pre-processing and post-processing approaches to focus on the infection ROIs.
